# Modifications in ankle dorsiflexor activation by applying a torque perturbation during walking in persons post-stroke: a case series

**DOI:** 10.1186/1743-0003-11-98

**Published:** 2014-06-09

**Authors:** Andreanne K Blanchette, Martin Noël, Carol L Richards, Sylvie Nadeau, Laurent J Bouyer

**Affiliations:** 1 Multidisciplinary Team in Locomotor Rehabilitation, Canadian Institutes of Health Research, Quebec, Canada; 2 Center for Interdisciplinary Research in Rehabilitation and Social Integration, 525 Wilfrid-Hamel Blvd, Quebec City G1M 2S8, Canada; 3 Department of Rehabilitation, Université Laval, 1050, ave de la Médecine, room 4472, Quebec City G1V 0A6, Canada; 4 Centre for Interdisciplinary Research in Rehabilitation of Greater Montreal, 6300 avenue Darlington, Montréal, QC H3S 2 J4, Canada; 5 School of Rehabilitation, Université de Montreal, Montreal, Canada

**Keywords:** Human locomotion, Motor control, Ankle dorsiflexors, Robotic device, Stroke, Adaptation, Rehabilitation

## Abstract

**Background:**

Results obtained in a previous study (Gait Posture 34:358–363, 2011) have shown that, in non-disabled participants, a specific increase in ankle dorsiflexor (Tibialis anterior [TA]) activation can be induced by walking with a torque perturbation that plantarflexes the ankle during the swing phase. After perturbation removal, the increased TA activation persisted temporarily and was associated with a more dorsiflexed ankle during swing. The objective of the present case-series study was to verify if these results can be reproduced in persons post-stroke.

**Methods:**

Six participants who sustained a stroke walked on a treadmill before, during and after exposure to a torque perturbation applied at the ankle by a robotized ankle-foot orthosis. Spatiotemporal gait parameters, ankle and knee kinematics, and the electromyographic activity of TA and Soleus were recorded. Mean amplitude of the TA burst located around toe off and peak ankle dorsiflexion angle during swing were compared across the 3 walking periods for each participant.

**Results:**

At the end of the walking period with the perturbation, TA mean amplitude was significantly increased in 4 of the 6 participants. Among these 4 participants, modifications in TA activation persisted after perturbation removal in 3 of them, and led to a statistically significant increase in peak dorsiflexion during swing.

**Clinical implications:**

This approach may be helpful to evaluate the residual adaptive capacity in the ankle dorsiflexors after a stroke and guide decision-making for the selection of optimal rehabilitation interventions. Future work will investigate the clinical impact of a multiple-session gait training based on this approach in persons presenting a reduced ankle dorsiflexion during the swing phase of walking.

## Background

After a central nervous system lesion (CNS), adaptation of the walking pattern to meet varied environmental demands, such as walking on a sandy beach or in snow, becomes more difficult. The neural control of walking involves complex interactions between different levels of commands. Central commands, from voluntary and automatic drive, predict the muscle activation pattern needed to perform the movement before its execution (feedforward control), while peripheral sensory inputs are involved in correction during movement execution (feedback control) and also in providing error feedback to prepare the next movement [1]. The CNS is constantly adjusting locomotor commands to the environmental constraints. A contribution of the cerebellum [2] and of the corticospinal tract [3,4] in locomotor adaptation have been proposed in the literature.

Over the last 20 years, paradigms consisting of walking in an experimentally controlled perturbing environment have been developed to evaluate this locomotor adaptive capacity [5-20]. A perturbation that induces a movement error during walking triggers a recalibration of central commands to adapt the muscle activation pattern to its presence. After perturbation removal, this recalibration persists temporarily and can lead to changes in lower limb kinematics (e.g. [10,11,13-15,17,19]). The perturbations used in these studies can be separated into 2 types: one that primarily affects a single lower limb (e.g. adding a weight on the leg or resisting a movement with a robotic device); and another that uses the walking surface to perturb interlimb coordination (e.g. split-belt treadmill).

A few recent studies have demonstrated that persons post-stroke are still able to adapt spatiotemporal gait parameters, such as step length, double support or single-limb support time to the presence of a perturbation during walking [21-24]. Adaptation of these parameters likely results from coordinated modifications in the activation of several muscle groups. To date, no experimental protocols have evaluated the residual adaptive capacity of a specific muscle group within a single limb while walking in a perturbing environment. This approach may be helpful to guide decision-making for the selection of optimal rehabilitation interventions in situations where the walking deficit is more localized.

A robotized ankle-foot orthosis that uses a hybrid drive system (electrohydraulic) was developed in our laboratory to apply phase-specific torque perturbations at the ankle joint during walking [25]. The effects of walking with a perturbation that targets the ankle joint during the swing phase on the locomotor pattern of non-disabled subjects were quantified in a previous study [19]. The purpose of the present case series was to evaluate ankle dorsiflexor residual adaptive capacity when persons post-stroke are exposed to a similar perturbation. Based on the results obtained in non-disabled subjects [19], we hypothesized that walking with a perturbation that plantarflexes the ankle during the swing phase would induce an increase in tibialis anterior (TA) activation in these subjects. We also hypothesized that such modifications would carry over after perturbation removal and lead to an increase in ankle dorsiflexion during the swing phase of walking. Preliminary results were published in abstract form [26].

## Methods

### Participants

Six individuals (S1 – S6) were recruited from the community to participate to the present experiment (Table 1). Inclusion criteria were as follows: participants had sustained a stroke more than 6 months prior to the study (chronic lesion), were discharged from rehabilitation, and were able to walk overground for at least 10 meters independently (i.e. without human assistance). Subjects were excluded if they had unstable health conditions or cardiovascular, pulmonary and/or cognitive deficits that could affect performance during the experiment. To reduce potential skin abrasions from the orthosis, persons having no light touch sensation on the foot and around the ankle were also excluded.

**Table 1:** Participant characteristics

**Participant**	**Gender, M/F**	**Age, year**	**Height, cm**	**Weight, kg**	**Lesion**	**Time post-lesion, month**	**Paretic side, R/L**	**Walking aid**
S1	M	69	168	82	Left ischemic stroke (frontoparietal)	61	R	Cane + AFO
S2	F	52	168	67	Left ischemic stroke (frontoparietal)	235	R	None
S3	M	70	168	70	Right ischemic stroke (frontoparietal)	27	L	Cane
S4	M	56	168	109	Right ischemic stroke (medulla oblongata)	42	L	Cane
S5	M	74	174	70	Left ischemic stroke (pons)	30	R	None
S6	M	79	170	68	Right ischemic stroke (frontal)	29	L	None

Participant demographic (gender, age, weight, height) and lesion characteristics (location, time post-lesion, paretic side, walking aid) are described. F: female; M: male; R: right; L: left; AFO: Ankle-foot orthosis.

### General protocol

For the *Evaluation of ankle dorsiflexor adaptive capacity* (see below), the test paradigm consisted of walking on a treadmill before, during and after exposure to a specific perturbation applied at the ankle. Before testing, each subject underwent a *Clinical examination* performed by a physical therapist to characterize ankle motor impairments. This protocol was approved by the Quebec City Physical Rehabilitation Institute human research review board and all subjects provided written informed consent prior to their entry in the study.

### Clinical examination

The *Clinical examination* documented the presence of motor impairments affecting the paretic ankle joint, such as dorsiflexor paresis, plantarflexor hypertonicity, or contractures [27,28]. Maximal passive ankle dorsiflexion was measured with a manual goniometer, with the participant laying supine with knee and hip joints at 0 degrees of flexion. Plantarflexor contractures were considered as present if passive dorsiflexion was less than or equal to 10 degrees [29]. Plantarflexor hypertonicity was evaluated with the Modified Ashworth Scale (MAS; [30]), which assesses ankle resistance to a velocity dependent stretch reflex using a 5-point nominal scale (ranging from 0 = “No increase in muscle tone” to 4 = “Affected part(s) rigid in flexion or extension”). Voluntary dorsiflexor strength was assessed using Daniels and Worthingham’s manual muscle testing grades (MMT; [31]), ranging from 5 = “Completes full range and holds against maximal resistance” to 0 = “No palpable contraction”.

### Evaluation of ankle dorsiflexor adaptive capacity

Participants walked with an electrohydraulic ankle-foot orthosis (EHO; [25]) on a treadmill at comfortable speed (range: 1.5-2.5 km/h) for 3 periods (duration ranging between 1 and 3 minutes): before, during and after walking with a perturbation. The CONTROL walking period was used to characterize individual baseline walking patterns (approximately 50 strides). It was followed by the PERTURBATION walking period, during which a torque perturbation was applied at the paretic ankle on every stride of walking. Participants were asked to: “Resist the force and try to walk normally”. The number of strides with a stable perturbation application was approximately 50. Modifications induced by walking with the perturbation were studied during this period. The POST walking period was used to document aftereffects following perturbation removal (approximately 50 strides). Participants walked continuously with the robotized orthosis during the 3 walking periods, looking at a target located 3.5 m straight ahead. When no perturbation was applied, the EHO actively compensated for friction and energy loss across the hydraulic circuit in order to minimize its effects on the subject's natural walking pattern (“active torque cancellation mode”; [25]). For security reasons, participants wore a harness attached to a ceiling-mounted suspension system (no weight support was provided) and lightly held the front handlebar of the treadmill. No walking aids (cane or ankle-foot orthosis) were used during the experiment. They were not informed of the exact time of transitions between walking periods to avoid anticipatory adjustments prior to these transitions.

**Figure 1:**
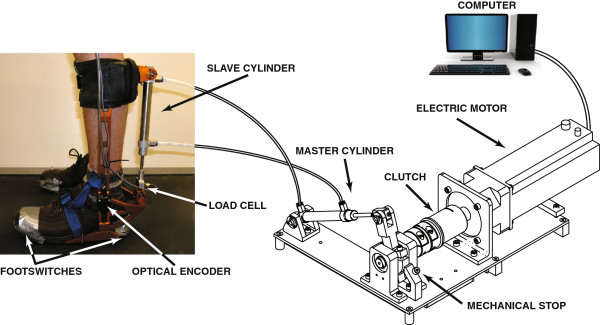
**Representation of the Electrohydraulic ankle-foot orthosis (EHO).** A line drawing of the drive system (on the right) and a picture of the ankle foot orthosis (on the left). The electric motor actuates a master cylinder (remote from the subject) that transfers the mechanical power through hoses to a slave cylinder attached to a custom-designed aluminum ankle-foot orthosis worn by the participant.

The perturbation was applied using a custom-designed EHO that uses a hybrid drive system combining the advantages of pneumatic, hydraulic, and electric systems (Figure 1; [25]). The EHO is characterized by lightweight actuators (pneumatic cylinders), a simplified force control algorithm (electric motor with PID controller), high power, and a short time constant (energy transfer by hydraulic fluid). The hybrid system is composed of an electric motor coupled to a hydraulic closed-loop system. The electric motor actuates a master cylinder (remote from the subject) that then transfers the mechanical power through hoses to a slave cylinder attached to a custom-designed aluminum ankle-foot orthosis worn by the participant. To optimize performance, pneumatic cylinders and hoses are filled with water instead of air to minimize compressibility and to improve system performance at higher frequencies.

The EHO enables the application of selected torque perturbations at the ankle joint at targeted moments of the gait cycle by rapidly switching from torque cancellation to torque generation. The torque generation mode is controlled in real time using: 1) a force signal from a load cell (Transducer Techniques Inc.) located at the lower extremity of the slave cylinder; 2) ankle angular position signals from an optical encoder (US Digital Inc.) located on the joint of the orthosis; and 3) signals from a footswitch placed under the heel as an indication of initial foot contact with the ground. More details can be found in Noël et al. [25].

In the present study, the perturbation consisted in applying a parabolic shape torque in the plantarflexion direction during the swing phase of walking, where the ankle has to move towards dorsiflexion in order to clear the toes off the ground. The exact timing and duration of the perturbation were adjusted for each participant on the basis of the ankle movements recorded during the baseline walking period. The perturbation was applied during the first half of swing phase (initial to mid-swing) to avoid disturbances in the preparation of foot contact with the ground. Perturbation onset was set approximately 50 ms after the initiation of swing phase (toe off) and the duration of the perturbation was fixed between 150 and 250 ms.

The following mathematical equation was used:

(1) $$\tau =K\;\left(-4u^{2}+4u\right)$$

In this equation, u = t/T where t = the onset time, and T = the total duration of the torque perturbation (seconds). K corresponds to the targeted peak amplitude (Nm). K was adjusted on an individual basis such that the smallest torque that induced a visible plantarflexion deviation in ankle trajectory was used.

Spatiotemporal gait parameters, ankle and knee kinematics and EMG data were recorded during this evaluation. Custom-made footswitches placed under the heel and big toe of both shoes were used to characterize spatiotemporal gait parameters. EMG activity of the tibialis anterior (TA) and Soleus (SOL) muscles of the paretic lower limb was recorded using disposable surface electrodes (KENDALL MEDI-TRACE 200, Covidien, Mansfield, USA) and a custom-made EMG amplifier. Electrodes were placed according to the Surface ElectroMyoGraphy for the Non-Invasive Assessment of Muscles (SENIAM) recommendations [32]. EMG signals were band-pass filtered (10–800 Hz) prior to acquisition, and then digitally filtered (zero-lag Butterworth 4th order filter; band-pass 10–450 Hz) before rectification. Knee and ankle angular displacements were measured with an electrogoniometer (Biometrics Ltd., Ladysmith, USA), and an optical encoder placed on the EHO (US Digital Inc., Washington, USA), respectively. Force applied by the slave cylinder was measured using a load cell (Transducer techniques, Temecula, USA). All signals were digitized at 1000 Hz.

### Data analysis

Custom-made software written in MATLAB (The MathWorks Inc., Natick, USA) was used for data analysis. Data recorded during the *Evaluation of ankle dorsiflexor adaptive capacity* were separated into individual strides based on footswitch signals, time-normalized to 100% for each stride, and then divided into 50 bins of equal width (2% of the gait cycle). Mean amplitude for each of these bins was reported for ankle and knee kinematics, torque, and EMG data.

To characterize the adaptive capacity of ankle dorsiflexors, TA mean amplitude of the burst located around toe off was calculated. A visual inspection of the mean TA activation profile in the presence of the perturbation enabled the identification of the TA burst location (around toe off) normally considered to be a concentric EMG activity burst involved in the dorsiflexion movement during swing. SOL mean amplitude was also measured in this zone to document potential coactivation when walking with the perturbation. Torque applied at the ankle was obtained by multiplying the linear force measured with the load cell by the length of the effective orthosis lever arm [25]. For each subject, the torque measured at baseline was subtracted from the torque recorded during the PERTURBATION walking period. Amplitude and position of peak added torque were then identified.

### Statistics

To evaluate ankle dorsiflexor adaptive capacity, the following 3 epochs were defined:

(a) “Baseline”: mean of the last 5 strides of the CONTROL period;

(b) “Adapted state”: mean of the last 5 strides of the PERTURBATION period;

(c) “Aftereffects”: mean of the first 5 strides of the POST period;

For each participant, one-Way ANOVAs were first used to determine if walking with the perturbation was having an overall effect on TA mean amplitude and maximal dorsiflexion during swing (intra-subject comparisons). T-tests were then used with Bonferroni correction to identify which epochs were different from baseline. Level of significance was set at 0.05.

## Results

### Ankle motor impairments in persons post-stroke

As demonstrated by the clinical assessment, persons post-stroke recruited in this study had varying levels of ankle motor impairments (Table 2).

**Table 2:** Clinical measurements characterizing ankle motor impairments (paretic ankle)

**CLINICAL MEASUREMENTS**			
**Participant**	**Passive dorsiflexion angle, degree**	**Plantarflexor hypertonicity, MAS score (0–4)**	**Dorsiflexor strength, MMT grade (0–5)**
S1	−10	4	1
S2	−2	3	2
S3	7	1+	2
S4	18	0	4
S5	−6	1	2
S6	12	0	5

Clinical measurements characterizing ankle motor impairments are described for the paretic side of each participant.

### Movement error induced by applying a perturbation at the paretic ankle during walking

**Figure 2:**
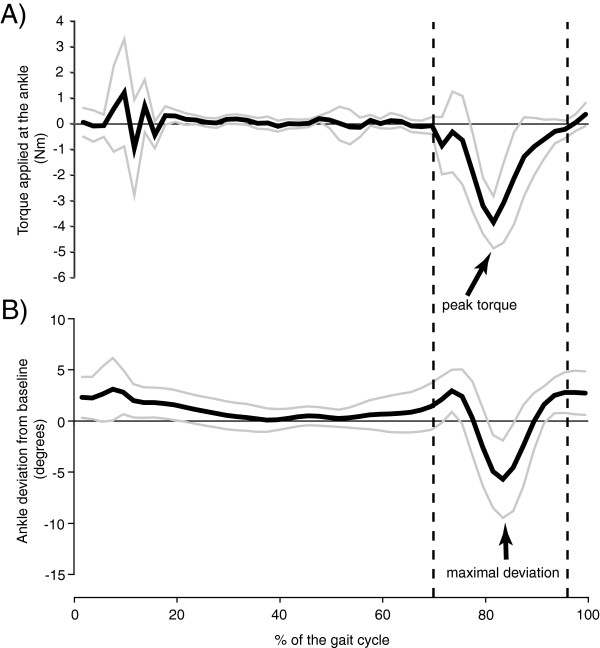
**Torque perturbation profile and deviation induced in ankle angular displacements during walking for one participant (S4).** In **A**, torque applied at the ankle during the walking period with the perturbation for one subject. In **B**, deviation induced in ankle angular displacements by walking with the perturbation for one subject. Black and grey lines represent the mean and SD (±) for all strides with the perturbation, respectively. Vertical dashed lines represent the onset and offset of the torque application.

Applying the perturbation at the ankle induced a deviation towards plantarflexion in the swing phase of walking, as demonstrated for one participant (S4) in Figure 2B. In this subject, a peak torque perturbation of 4.7 ± 0.6 Nm, located at 82.2 ± 2.2% of the gait cycle (Figure 2A) produced an ankle deviation of 6.9 ± 3.5 degrees at 83.6 ± 2.0% of the gait cycle (Figure 2B).

As mentioned under Methods, the amplitude and position of the torque perturbation were adjusted on an individual basis due to the inter-subject differences in the baseline walking pattern. Perturbation characteristics are presented in Table 3. As the goal of the experiment was to walk with a stable torque perturbation for 50 strides (actual 53.2 ± 8.6 strides), and considering that it took between 0 and 38 strides to adjust peak torque amplitude, participants walked for a varying number of strides while being actually perturbed (range: 50–111 strides).

**Table 3:** Torque perturbation characteristics

**Participant**	**Amplitude of peak torque. Nm**	**Position of peak torque, % of the gait cycle**	**Maximal plantarflexion deviation during swing, degrees**
S1	1.9 ± 0.5	80.1 ± 2.8	2.4 ± 3.0
S2	3.0 ± 0.6	82.8 ± 2.5	15.6 ± 3.2
S3	3.2 ± 0.8	80.8 ± 2.5	5.6 ± 3.3
S4	4.7 ± 0.6	82.2 ± 2.2	6.9 ± 3.5
S5	2.5 ± 0.5	76.5 ± 2.0	4.2 ± 1.3
S6	3.0 ± 0.4	76.2 ± 4.6	2.1 ± 1.8

Torque perturbation application is characterized by the following variables for each participant: amplitude and position of the peak torque, as well as maximal ankle plantarflexion deviation induced by the perturbation (mean ± SD).

### Ankle dorsiflexor adaptive capacity to a perturbation during walking in persons post-stroke

**Figure 3:**
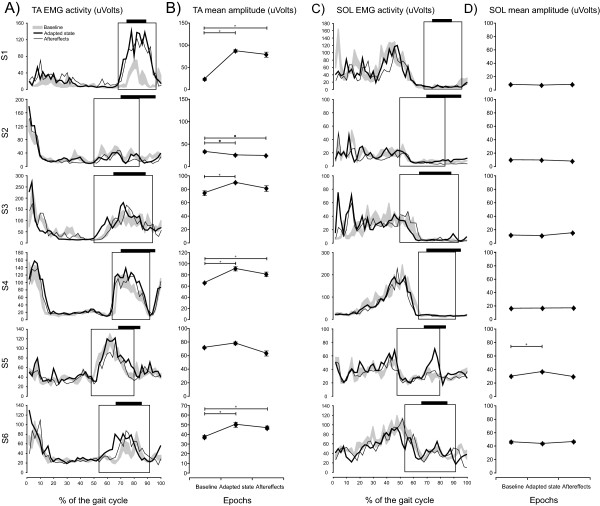
**Effects of walking with the perturbation on ankle muscle groups for each participant.** In **A** and **C**, modifications in Tibialis anterior (TA; panel **A**) and Soleus (SOL; panel **C**) activation profile induced by walking with the perturbation for each subject. Three curves representing filtered and rectified EMG activity are superimposed: baseline (grey trace; mean ± standard error of the mean [SE]), adapted state (thick black line), and aftereffects (dashed black line). Each rectangle area represents the section of the gait cycle further analyzed and presented in Panels **B** and **D**. In **B** and **D**, the mean TA **(**panel** B)** and SOL **(**panel** D)** amplitude (uVolts) during the stance-to-swing burst is compared between baseline, adapted state, and aftereffects for each subject. Horizontal black bar over each EMG activity panel represents the exact location of torque application. Error bars represent the SE. * = significant increase with a p < 0.05; + = significant decrease with a p < 0.05.

In non-disabled persons, a previous study has shown that walking with this perturbation produced an increase in TA activation around toe off [19]. Similar results occurred in 4 of 6 participants post-stroke. In these participants, a significant increase in TA mean amplitude in the swing phase, ranging from 21.0% to 288.6% (% of baseline; S1: p < 0.001; S3: p = 0.011; S4: p < 0.001; and S6: p < 0.001), was observed at the end of the PERTURBATION walking period (Figure 3A and B). This effect was not present in S2 or S5, however. In S2, the baseline TA activation during swing was so low that the usual stance-to-swing transition burst was undistinguishable from background noise, and changes in TA activation during swing were thus difficult to interpret. In S5, walking with the perturbation did not have a statistically significant impact on TA mean amplitude in the specific zone of the gait cycle (p = 0.222).Walking with the perturbation did not induce concomitant activation of the antagonist muscle (SOL) during the swing phase in 5 of the 6 participants (Figure 3C and D; S1: p = 0.393; S2: p = 0.056; S3: p = 0.064; S4: p = 0.685; S6: p = 0.432). In the other participant (S5), SOL mean amplitude increased significantly from 28.8 ± 0.8 μV at baseline to 35.7 ± 1.0 μV in adapted state, corresponding to an increase of 24.0% (p = 0.001).

**Figure 4:**
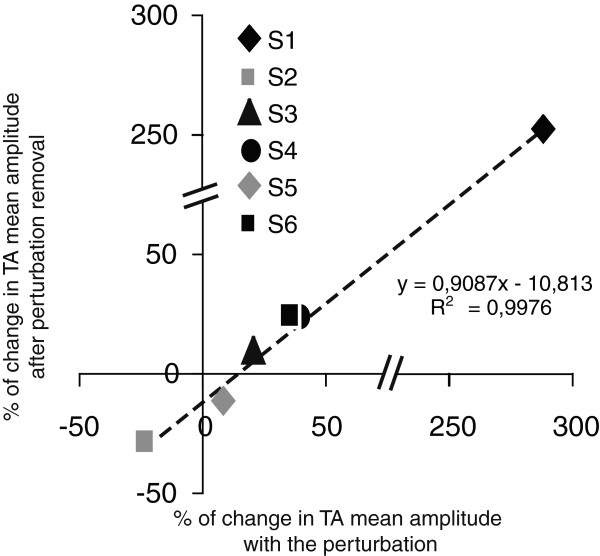
**Relationship between changes in TA mean amplitude observed with the perturbation and after its removal.** Modifications in TA mean amplitude observed after perturbation removal (aftereffects;% of baseline) in relation to modifications observed with the perturbation (adapted state;% of baseline) for each participant. Each symbol represents a single participant. Dashed line consists of a 2-parameter linear fit (R^2^ = 0.9976).

Among the 4 participants showing an increased TA activation during the PERTURBATION period, these effects carried over after perturbation removal in 3 of them. Persisting increases in TA mean amplitude in the first strides after removing the perturbation ranged between 24.0% and 251.5% (% of baseline; Figure 3). These aftereffects were significantly different from baseline (S1: p < 0.001; S4: p = 0.013; S6: p = 0.007). The relationship between changes in TA activation obtained during and after exposure to the perturbation can be observed in Figure 4. This relationship was nearly linear, as demonstrated by a coefficient of determination value (R^2^) of 0.9976. It indicates that more than 99% of the variance in the modifications in TA amplitude observed after perturbation removal may be explained by the modifications observed with the perturbation.

**Figure 5:**
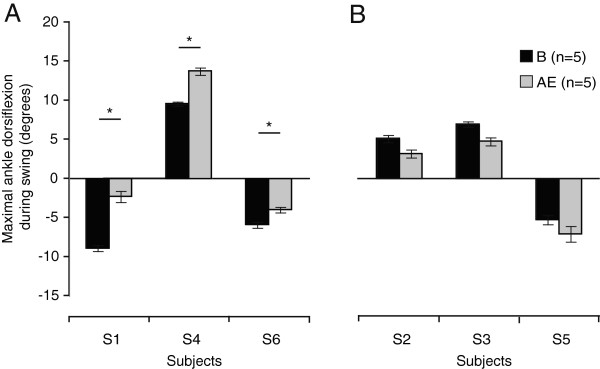
**Effects of walking with the perturbation on maximal ankle dorsiflexion during swing after perturbation removal.** Maximal ankle dorsiflexion during swing (in degrees) is compared between baseline (B; black bar) and aftereffects (AE; grey bar) for each participant. Panel **A** includes the 3 participants showing aftereffects in TA mean amplitude, while Panel **B** includes the others. Error bars represent the SE. * = significant increase (p < 0.05).

As mentioned above, the number of strides taken with the perturbation varied across participants (between 50 and 111 strides). However, no relationship was found between exposure duration and aftereffects magnitude (data not shown).These aftereffects in TA activation led to modifications in ankle angular displacement. Significant increases of 6.6° (p < 0.001), 4.2° (p < 0.001), and 1.9° (p = 0.006) in maximal dorsiflexion during the swing phase when compared to baseline can be observed after removing the perturbation, in S1, S4, and S6, respectively (Figure 5).

## Discussion

The purpose of the present case series was to evaluate ankle dorsiflexor residual adaptive capacity in persons who had sustained a stroke by having them walk on a treadmill with an electrohydraulic ankle-foot-orthosis that applied a torque perturbation at the ankle joint in the swing phase of the gait cycle.

### Walking with the EHO

It was first necessary to investigate the feasibility of using the EHO to apply a perturbation at the ankle during the swing phase of walking in persons post-stroke. Despite the presence of ankle motor impairments associated with their lesions, participants were able to clear the ground and to walk safely with the EHO. When questioned about their reactions to walking with the EHO, participants reported mild discomfort due to the added mass and the limitation in medio-lateral ankle movements. The aluminum structure was optimized to combine low weight properties with the capacity to sustain sufficient torques. The total mass of the EHO without the shoe is 1.70 kg [25]. This weight seems reasonable considering that adding a unilateral weight of 1.82 kg or less does not result in a significant change in walking speed or alter oxygen consumption in non-disabled persons [33]. The results of the present study show that persons post-stroke can safely walk with the EHO.

### Adaptive capacity to a perturbation during walking in persons post-stroke

The present study evaluated the dorsiflexor adaptive capacity in 6 persons post-stroke by walking with a perturbation that plantarflexed the ankle during the swing phase. Similar to the results obtained in non-disabled individuals [19], walking with this specific perturbation led to an increased TA activation in 4 of 6 participants, indicating that these individuals still had the capacity to adapt dorsiflexor activation to changes in environmental demands. Furthermore, the modification in TA activation carried over after perturbation removal in 3 of these 4 participants. These results are consistent with a central recalibration of the motor commands (feedforward mechanisms; [34]).

The absence of aftereffects in S3 suggests that modifications in TA activation observed in the presence of the perturbation did not involve a central recalibration of the motor commands. For this particular subject, the increased TA activation in the adapted state may therefore have been due to modifications in the sensory afferent pattern resulting from the action of the perturbation on the lower limb while walking. By moving the ankle towards plantarflexion during the swing phase, the perturbation may have modified spindle afferent discharge as a result of a different muscle elongation/shortening pattern during walking [35]. Moreover, skin afferent discharge may also have been modified in the presence of the perturbation. When the perturbation was applied, the shoe pushed on the dorsal surface of the foot to move the ankle towards plantarflexion. Considering that feedback mechanisms are involved in the neural control of locomotion [1,36], this modified sensory afferent pattern likely participated to the reshaping of TA muscle activation during walking.

Targeted modifications in TA activation were not obtained in all participants, however. In the remaining 2 participants, no significant increase was observed in TA activation during swing. While S2 did not modify activations of any of the recorded muscles to counteract the effects of the perturbation, S5 increased SOL activation during swing. The resulting TA-SOL coactivation around the ankle joint may have been used to minimize the effects of the perturbation on his walking pattern. The central nervous system is known to adapt the mechanical impedance (stiffness) of the joint by modulating coactivation of antagonist muscle groups during perturbed reaching movements [37-40]. A similar strategy may be adopted in the lower limb when post-stroke persons are walking in a perturbing environment.

Other features of the adaptive capacity in the presence of a perturbation after a stroke have been previously studied [21,22,24,41]. Indeed, Reisman et al. [21,22] have demonstrated that persons post-stroke are able to adapt their step length and double support time of the paretic limb to different belt speed relationships (split-belt locomotion). Moreover, modifications in walking symmetry were obtained in a recent study following adaptation to a perturbation that resisted lower limb forward movement with a system of pulleys and weights [24]. These results suggest that, after a cerebral lesion, the impaired CNS is still capable of adapting spatiotemporal gait parameters to a perturbing environment. However, modifications of these inter-limb coordination parameters may involve compensations rather than actual adaptation in some participants. Contrary to these studies, the approach presented here enables the assessment of residual adaptive capacity in a targeted muscle of a single limb during the swing phase, thereby limiting possible contralateral limb compensations. That being said, we are aware that it is very likely that inducing modifications in a muscle group during walking may have consequences on the dynamic control of the whole limb and therefore also affect other muscle groups.

### The impact of ankle motor impairments on dorsiflexor adaptive capacity

Results of the present work also showed that chronic motor impairments affecting the paretic ankle joint are not good predictors of a lack of adaptive capacity in TA muscle activation when walking with the perturbation, as demonstrated by S1 who presented the most pronounced ankle motor impairments of all participants. In this specific participant, clinical measurements indicated a severe plantarflexor hypertonicity with a marked contracture (ankle passive dorsiflexion did not reach the neutral position), as well as an inability to produce a voluntary dorsiflexion movement. Despite the severity of his ankle motor impairments, S1 demonstrated a very good dorsiflexor adaptive capacity when walking with the perturbation. The lack of correlation between impairments and adaptive plasticity during split-belt locomotion in stroke patients was also reported in Reisman et al. [21]. Further work with larger sample sizes is needed to examine the relationship between specific motor impairments or walking deficits and adaptive capacity.

### Clinical implications

Several authors have suggested that walking in a perturbing environment could be helpful in the rehabilitation of persons with walking limitations following CNS lesions (e.g. [19,21,22,24,42-46]). The premise is that amplifying a walking deficit produces a larger movement error that in turn may trigger a recalibration in central commands to ultimately correct it. As an example, a few protocols voluntarily heighten the asymmetrical locomotor pattern of persons post-stroke by walking with different belt speed relationships (split-belt locomotion; [21,22]). Aftereffects resulting from the adaptation to this perturbing environment then improved walking symmetry.

The demonstration that inducing a plantarflexion deviation during walking triggers a central recalibration of the motor commands that leads to an increase in ankle dorsiflexion after perturbation removal supports the potential of such an approach to improve walking limitations. This innovative approach of inducing “angular” movement error, enables the targeting of a specific joint and/or phase-specific motor impairment during walking, such as foot drop (see below) that cannot be addressed with systems like split-belt treadmills.

To date, only a few studies have documented the long-term effects of gait training based on walking in a perturbing environment to improve specific walking disabilities resulting from a cerebral lesion [44,47]. More specifically, multiple sessions of walking with a weight attached on the paretic leg have been reported to lead to improvements in functional ambulation of persons post-stroke, particularly in locomotor activities requiring knee and hip flexion, such as stair climbing [47]. Furthermore, a better step length symmetry of a person post-stroke was reported after split-belt treadmill training [44].

Considering that an increase of 5 degrees in dorsiflexion range of movement is usually considered as clinically significant [48], some may argue that statistically significant increases in ankle dorsiflexion obtained in the present study (6.6, 4.2 and 1.9 degrees) might not have a clinical impact on locomotion. However, these effects were induced by a single session of walking with the perturbation (duration ranging between 1 and 3 minutes). It is reasonable to hypothesize that persons presenting a reduced ankle dorsiflexion during the swing phase of walking (“foot drop”) may benefit from gait training (multiple sessions) based on this approach. The potential clinical impact of an increased dorsiflexion during the swing phase of walking is likely related to an increase in toe clearance that in turn will facilitate forward body progression and reduce the risk of tripping or falling.

Before testing a locomotor training program based on walking with this specific perturbation on persons post-stroke, it is important to address whether the modifications in TA activation transfer to overground walking. To our knowledge, only one study has documented the transfer of aftereffects induced by a perturbation during treadmill walking to overground walking in persons post-stroke [22]. In that study, a partial transfer of the aftereffects induced by walking on a split-belt treadmill to overground walking occurred in non-disabled and post-stroke subjects, suggesting that some aspects of the neural control of overground walking are shared with split-belt treadmill walking. The authors suggest that the transfer would be larger if more characteristics were common to both environmental contexts. Further work is needed to document the transfer of the aftereffects resulting from adaptation to a perturbation such as that used in the present study to overground walking.

### Study limitations

The demonstration of the potential of this approach to evaluate the residual adaptive capacity in the ankle dorsiflexors after a stroke in only six subjects could be considered a study limitation. As demonstrated, however, the evaluation of the adaptive capacity must be made on an individual basis using intra-subject comparative analysis. This therefore greatly reduces the number of subjects needed for a case-series study, like this one.

Sensory impairments were not documented in details in the present study. Only participants with no light touch sensation on the foot or around the ankle were excluded to avoid pressure sores. Considering the contribution of sensory inputs to the control of walking [1,36], it is possible that they would correlate better with adaptive capacity. While results presented in the literature vary with regard to the role of sensory impairments in motor adaptation (see [21,49]), we suggest that sensory impairments should nevertheless be considered in future studies. Current methods to test proprioceptive deficits at the lower limb are often based on simple tests such as identifying the direction of passive movement of the big toe [21]. As it is reasonable to believe that adaptation while walking with a perturbation may be influenced by the capacity to detect errors in lower limb joint position, further work is needed to first develop a valid and reliable proprioception measurement tool for person with motor deficits and then to investigate the relationship between proprioceptive deficits and adaptive capacity.

While no major limitations were found in using the EHO to apply perturbations at the ankle of persons post-stroke during treadmill walking, the need to use customized shoes restricted somewhat our subject selection. Indeed, persons having no light touch sensation on the foot and around the ankle had to be excluded to avoid potential skin damage when the EHO was worn. An improvement would be to modify the design of the EHO to allow personal shoes to be used.

## Competing interests

The authors declare that they have no competing interests.

## Authors’ contributions

AKB participated in the design of the study, was responsible for data collection, carried out the data analysis, performed the statistical analysis, and drafted the manuscript. MN was responsible for the software modifications and control of the robotized orthosis during data acquisition, participated in data collection, and helped to draft the manuscript. CLR contributed to the design of the study and helped to draft the manuscript. SN contributed to the design of the study and revised the manuscript. LJB conceived the study, participated in its design, coordination and analysis, and helped to draft the manuscript. All authors read and approved the final manuscript.
